# Clinical Evaluation of Preventive Effect of Fissure Sealants on Initial Carious Lesion of Permanent Mandibular Molars Pretreated with and without Fluoride Varnish by Fluorescence Camera

**DOI:** 10.5005/jp-journals-10005-1491

**Published:** 2018-04-01

**Authors:** Madhagudanahalli S Lakshmi, Kudlapur T Srilatha, Bhojraj Nandlal, Seema Deshmukh

**Affiliations:** 1 Senior Lecturer, Department of Pediatric and Preventive Dentistry, JSS Dental College & Hospital, Mysuru, Karnataka, India; 2 Professor and Head, Department of Pediatric and Preventive Dentistry, JSS Dental College & Hospital, Mysuru, Karnataka, India; 3 Professor, Department of Pediatric and Preventive Dentistry, JSS Dental College & Hospital, Mysuru, Karnataka, India; 4 Reader, Department of Pediatric and Preventive Dentistry, JSS Dental College & Hospital, Mysuru, Karnataka, India

**Keywords:** Fluorescence, Fluoride varnish, Pit and fissure sealant.

## Abstract

The important armamentarium in the present day scenario against caries prevention is considered to be a combination of preventive methods. The present study was conducted to evaluate the preventive effect of pit and fissure sealant pretreated with fluoride varnish on initial carious lesion by fluorescence camera (FC).

**How to cite this article:** Lakshmi MS, Srilatha KT, Nandlal B, Deshmukh S. Clinical Evaluation of Preventive Effect of Fissure Sealants on Initial Carious Lesion of Permanent Mandibular Molars Pretreated with and without Fluoride Varnish by Fluorescence Camera. Int J Clin Pediatr Dent 2018;11(2):89-93.

## INTRODUCTION

Recent advancements in the field of prevention encourage the adoption of a combination of preventive programs. Paradoxically, lifelong exposure to different sources of fluoride is thought by many to be largely responsible for a change in the pattern and progression of dental caries.^1,2^ Pit and fissure sealants have been considered an important adjunct to oral health care strategies and fluoride therapy in preventing occlusal carious lesion. And topical fluoride therapy in the form of varnish, which forms calcium fluoride, will act as a slow releasing agent to enhance remineralization and make the enamel more resistant to acid dissolution, thus inhibiting demineralization that may occur if the sealant is lost between the recall visits.^3^ Hence, the diagnosis plays a major role. There are various techniques of rem-ineralization, yet their implementation has been strongly hindered because of the inability to detect early lesions. The advent of newer diagnostic aids has offered the possibility of detection of such lesion and thus investigate preventive and reversal therapies. So, currently FC system seems to be the optical method with the highest potential for diagnosis and quantification of the earliest carious changes in the enamel.^2^

The aim of the study was to evaluate the preven-five effect of fissure sealant on initial carious lesion of the permanent mandibular molars pretreated with and without fluoride varnish and to correlate the preventive effect of fissure sealant on the initial carious lesion of the permanent mandibular molars with marginal adaptation, retention, and caries progression using FC.

## MATERIALS AND METHODS

The sample consisted of 24 subjects aged 8 to 9 years selected based on the exclusion and inclusion criteria from a residential school. Written consent from the parents and consent from the students included in the study were also taken before commencing the study. Ethical clearance was obtained from the ethical committee, JSS Dental College and Hospital under JSS University, Mysuru, Karnataka, India. The residential schoolchildren were included in the study to maintain the uniformity of the data with uniform dietary patterns. The null hypothesis of the study is that pretreatment with fluoride varnish will not have any effect on the sealant application.

### Inclusion Criteria for Application of Fissure Sealants

Subjects with first permanent mandibular molars having International Caries Detection and Assessment System (ICDAS) scores of 1, 2, and 3 were selected for the study. Bite wing radiographs were taken to rule out proximal caries. After having selected the subjects based on the above-mentioned criteria, baseline fluorescence examination was done and only those subjects with fluorescence scoring of 1.0 to 1.5 were included in the study.

### Sample Size Determination

Determining at power of 80%, test size of 5% with mean reduction in the caries score of 1.1 in conventional technique and expected reduction in modified technique 1.2, the sample size of 12 in each group was arrived at.

## ALLOCATION PROCEDURE

Block randomization technique was used with concealed allotment (sequentially numbered, opaque, sealed envelopes) following generation of random allocation sequence. Allocation ratio was 1:1. The random allocation was generated by the investigator who also allotted the participants to two study groups. Each group consisted of 12 subjects with one group receiving only pit and fissure sealant (control called as sealant group) and the other group receiving fluoride varnish pretreatment followed by pit and fissure sealant application after 48 hours (varnish-pretreated group). Here, the double-blinding technique was followed in which the investigator did not know to which group the sealant was applied. However, a single investigator was involved in recording the fluorescence scores and images preoperatively and at subsequent interval periods.

Fissure sealants and fluoride varnishes were administered: nanofilled fissure sealant (Grandioseal, VOCO, Germany) and fluoride varnish (Bifluoride varnish, VOCO). Before application of fissure sealant, oral hygiene was assessed by application of plaque score. Turesky’s modification of Quigley-Hein plaque index and appropriate oral hygiene practices were advised for the subject. Another 12 subjects who were assigned for varnish-pretreated group received fluoride varnish application followed by pit and fissure sealant application after 48 hours. All the above procedures were done under rubber dam isolation.

## FLUORESCENCE ANALYSIS

Fluorescence analysis of the lesions was conducted by a single examiner using fluorescence device (FC, Duerr Dental, Vistaproof, Germany^1^) which uses the DDview software to analyze the carious lesions. This recently devised FC emits blue light at 405 nm, and records fluorescence from the teeth as digital images. This software shows the region of the teeth that emits fluorescence ranging from 0 to 3 corresponding to the lesion severity and calculated as the intensity ratio of the red and green fluorescence. Fluorescence reading indicates the change in the value of fluorescence. Increase in the reading indicates loss of fluorescence which corresponds to demin-eralization, while decreases in the reading correspond to remineralization.

## RELOCATION OF THE SAME HIGHEST SCORE POINT

**Figs 1A to D:**
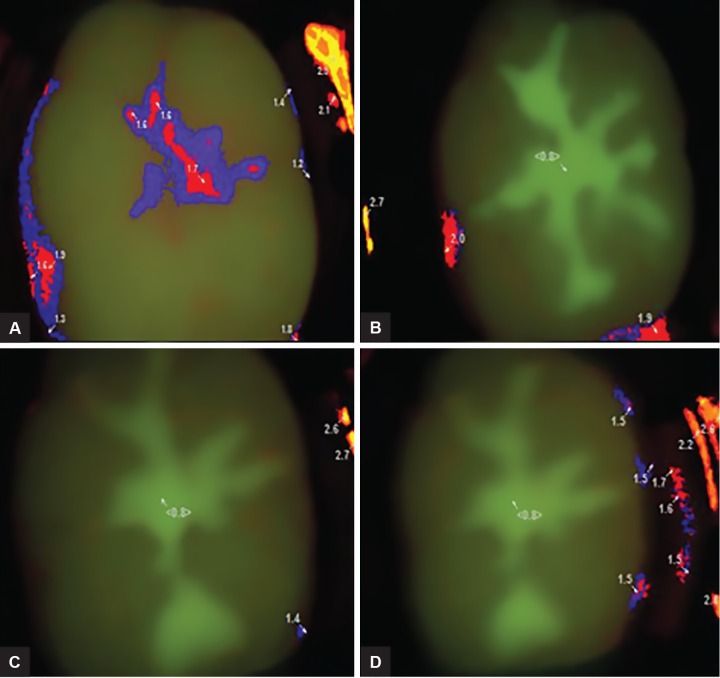
Relocation of the highest score point from the X- and Y-axis coordinates

The X-axis and Y-axis coordinates of the highest score point were noted down as given by the image software analyzer (DDWIN) at baseline (before application of pit and fissure sealant and after application of sealant in the subsequent time interval). Fluorescence examination scores with the same values of the X-axis and Y-axis coordinates were recorded. The fluorescence image of the sample was compared with the previous image taken at the baseline to avoid any magnification error before recording the highest score point (Fig. 1).

### Outcome Measurement

The subjects were evaluated at 3rd and 6th month follow-up where the retention, marginal adaptation, and caries and fluorescence images were recorded. The software of fluorescence system can process the image to provide user the quantitative parameters, such as lesion area, lesion depth, and lesion volume.

These parameters can detect and differentiate the lesions at very early stages, and make the fluorescence system more sensitive to changes of caries over time. The image can be stored for longitudinal study and be used as patient motivators in a preventative practice.

Retention of the sealant was checked by attempting to dislodge the sealant with the help of an explorer tip and the following scoring criteria were used: 1 = completely retained, 2 = partial loss, and 3 = total loss.

For clinical scoring for caries done with the help of air spray and isolating the teeth with rubber dam isolation, the following scoring criteria were used: 0 = absence of caries and 1 = presence of caries.

Clinical scoring for marginal adaptation was done by running the tip of the explorer around the margins of the sealant and checking the presence of catch, and the following scoring criteria was used: 1 = no defect, 2 = slight catch, 3 = moderate catch, 4 = slight crevice, and 5 = extensive crevice.

The subjects were evaluated at the 3rd and 6th month follow-up where the retention, marginal adaptation, and caries and fluorescence images were recorded. The software of FC systems can process the image to provide user quantitative parameters, such as lesion area, lesion depth, and lesion volume. These parameters can detect and differentiate the lesions at very early stages, and make the FC system more sensitive to changes of caries over time. The image can be stored for longitudinal study and be used as patient motivators in a preventative practice.

Retention of the sealant was checked by attempting to dislodge the sealant with the help of an explorer tip and the following scoring criteria were used: 1 = completely retained, 2 = partial loss, and 3 = total loss.

For clinical scoring for caries done with the help of air spray and isolating the teeth with rubber dam isolation, the following scoring criteria were used: 0 = absence of caries and 1 = presence of caries.

Clinical scoring for marginal adaptation was done by running the tip of the explorer around the margins of the sealant and checking the presence of catch, and the following scoring criteria were used: 1 = no defect, 2 = slight catch, 3 = moderate catch, 4 = slight crevice, and 5 = extensive crevice.

### Statistical Analysis

All data were registered on specific forms that were optically transformed into the IBM Statistical Package for the Social Sciences version 18 software for descriptive statistics. Differences between the groups at baseline and follow-up were tested by independent sample t-test and chi-square tests; p-values < 0.05 were considered statistically significant.

## RESULTS

The mean duration between the baseline and the follow-up was 6 months for both the groups. There were no dropouts in the study. The children of both groups were balanced and comparable at the baseline with reference to age, sex, and general health. There was no significant increase in the mean fluorescence score of both groups at the end of 6 months with p > 0.05, indicating that there is no progression of the carious lesion (Table 1).

In terms of retention and marginal adaptation, it was observed that the sealant group (control group) showed no defect in 75% of the cases and slight catch in 25% of the cases at 3rd and 6th month and in varnish-pretreated group showed slight catch in 66.6% of the cases at the end of 3rd and 6th month and moderate catch in 8.33% of the cases at the end of 6th month; however, no new carious lesions were observed, which suggests that the fluoride varnish application did not jeopardize the effectiveness of the sealant (Tables 2 and 3).

**Table 1:** **Table 1:** Intergroup comparison of quantitative light-induced fluorescence highest score point between sealant and varnish-pretreated group

*Period*		*Group*		*Mean*		*SD*		*t-value*		*p-value*	
Baseline		Sealant		1.50		0.20		0.37		0.72 NS	
		Varnish pretreated		1.54		0.24					
Postsealant		Sealant		0.85		0.05		1.25		0.22 NS	
		Varnish pretreated		0.83		0.04					
3rd month		Sealant		0.94		0.16		1.37		0.18 NS	
		Varnish pretreated		0.87		0.06					
6th month		Sealant		1.02		0.23		1.53		0.14 NS	
		Varnish pretreated		0.92		0.07					

NS: Nonsignificant; SD: Standard deviation

**Table 2:** **Table 2:** Intergroup comparison of retention

		*3rd month*				*6th month*			
		*Control*		*Study*		*Control*		*Study*	
Complete retention		91.6% (11)		83.3% (10)		75% (9)		75% (9)	
Partial loss		8.33%		16.6%		25% (3)		25% (3)	
Total loss		0% (0)		0% (0)		0% (0)		0% (0)	

### DISCUSSION

Dental caries, although it has declined in recent years, seems to be the most prevalent oral disease in India.^2^ Since primary prevention with minimum effort and maximum results can be instituted in pediatric dental practice, fluoride treatment has become an integral component of preventive programs. Occlusal surfaces are least protected by fluoride, thus rationalizing the use of sealant as a complementary procedure. As the sealants occluded the fissures, questions regarding whether the caries could progress beneath the sealant soon arose. After a number of studies, a clear answer was obtained that, when the sealant is placed over an incipient carious lesion, caries does not progress, provided the sealant remains intact. Studies by Swift^4^ and Going et al^5^ have concluded that the evidence is strong that caries-active lesions become caries inactive beneath the intact sealant. Though the application of pit and fissure sealant placed over an incipient caries lesion has shown to arrest the progress of caries, it is again subjected to retention of sealant.^4^ Since a number of different approaches to prevent dental caries are now available, fissure sealants are only a part of the program of prevention, but an important armamentarium against dental caries is combination of preventive programs. The study conducted by Whelton and O’Mullane^6^ suggested the potential effectiveness of combinations of preventive methods and concluded that the most promising combination program currently appears to be the use of fluoride with fissure sealing. In order to implement potential treatment, attention of clinicians and researchers has been drawn to the detection, quantification, monitoring, and, ultimately, diagnosis of early carious lesions.^7,8^

Fluorescence technology has offered the research community the ability to further elucidate the carious process and study the effect of new therapies and products on the demineralization/remineralization dynamic. The study conducted by Heinrich-Weltzein et al^9^ shows that FC can be useful to detect imperfect margins by a reddish fluorescence, characterizing a microleakage and inferior quality of the interface between filling or sealant material and tooth substance. Hence, FC may support the decision-making process on whether a restoration should be controlled or replaced. Fluorescence camera may be a substantial aid to assess the quality of sealants and a diagnostic aid when judging a surface for caries prior to sealant application. Though the fluorescence method is said to be one of the nonconventional detection methods for early incipient lesion, it is subject to confounding effects of saliva, drying time, angulations, and stain.^10^

**Table 3:** **Table 3:** Intergroup comparison of marginal adaptation

		*3rd month*				*6th month*			
*Scoring criteria*		*Control*		*Study*		*Control*		*Study*	
Slight catch		83.3% (10)		75% (9)		75% (9)		66.6% (8)	
Moderate catch		16.6% (2)		25% (3)		25% (3)		25% (3)	
Slight crevice		0%		0%		0%		8.33% (1)	
Extensive crevice		0%		0%		0%		0%	

The results showed that the mean fluorescence score from the baseline has significantly reduced at the end of third and sixth month in both sealant- and varnish-pretreated groups and there is more significant decrease in the fluorescence score in the pretreated group, suggestive of anticariogenic effect.

In the present study, at the end of the 6th month, since more than 60% of the sealants were intact, it suggests that fluoride varnish application did not jeopardize the effectiveness of sealant, which goes in accordance with the study conducted by El-Housseiny and Sharaf.^3^

The study conducted by Went et al^11^ has shown that the partial retention of the sealant is often considered a success, because if some part of the sealant is missing in the fissures, there is still enough resin in the deeper part to prevent caries.

Another study conducted by Garcia-Godoy et al^12^ has proved that there is highest bond strength in fluoride-treated teeth and has suggested that the bonding is not adversely affected by the application of fluoride prior to etching.

At the end of the study, within the limitations, we suggest that though the sealants are considered a more primitive preventive treatment, periodic review and replacement are recommended and therefore, pretreatment with fluoride varnish is recommended, as it provides prevention against caries, if the sealant is lost in between the visits.^13,14^

## CONCLUSION

Irrespective of the extensive preventive regimens being followed by the pediatric dentists, dental caries remains the most common childhood disease. This indicates the urgent need for improvised methodologies for caries diagnosis and prevention. This study attempts to utilize a combination of different preventive modalities traditionally being used for prevention of dental caries combined with advanced caries diagnostic aid, which would favor early identification. Thus, the study indicates that the combination of various traditionally available preventive therapy would be more beneficial than routine methods.
